# A case report on ileal trichobezoar: a surgical emergency in a 7-year-old

**DOI:** 10.1097/MS9.0000000000005761

**Published:** 2026-10-08

**Authors:** Sudesh Lamsal, Adina Bataju, Chiran Bhakta Bista, Sonali Shrees Magar, Riya Jaiswal, Sudha Karki, Aashutosh Pokharel

**Affiliations:** a Department of Surgery, Shree Birendra Hospital, Kathmandu, Nepal; b Nepalese Army Institute of Health Sciences, Kathmandu, Nepal

**Keywords:** bezoars, case report, hair ball, trichobezoar, trichophagia

## Abstract

**Introduction and importance:**

Trichobezoars are rare gastrointestinal foreign bodies formed from ingested hair, most commonly associated with trichotillomania and trichophagia. While they typically occur in adolescent females, their presentation as an isolated small-bowel obstruction in younger children is exceptionally uncommon and poses significant diagnostic challenges, highlighting the need for early clinical awareness.

**Presentation of the case:**

A 7-year-old female with a history of trichotillomania presented with a 5-day history of generalized abdominal pain, vomiting, and absolute constipation. Examination revealed abdominal distension, guarding, and sluggish bowel sounds. The patient’s workup suggested a heterogeneous intraluminal mass with a transition point consistent with a trichobezoar. A 9–10 cm ileal trichobezoar, 20 cm proximal to the ileocecal junction, was removed via enterotomy. The patient made a full recovery.

**Clinical discussion:**

Trichobezoars in young children present atypically and require a high clinical suspicion. CT is the most sensitive diagnostic modality, and surgery remains the definitive treatment for obstructing bezoars.

**Conclusion:**

Trichobezoars should be considered in pediatric bowel obstruction, particularly in females with subtle behavioral clues. Early diagnosis, timely surgical intervention, and psychological evaluation are essential to prevent recurrence.

## Introduction

A bezoar is an accumulation of indigestible material within the gastrointestinal tract that can lead to varying degrees of gastric obstruction. Bezoars can be classified as phytobezoars, trichobezoars, lactobezoars, and pharmacobezoars based on their composition. Trichobezoars are composed primarily of hair and represent a rare medico-surgical entity, with a prevalence ranging from 0.4% to 0.6%^[^1^]^.

HIGHLIGHTS

- Ileal trichobezoar is a rare cause of small bowel obstruction in children.
- A 7-year-old girl presented with abdominal pain, vomiting, and absolute constipation.
- Contrast-enhanced CT showed a heterogeneous intraluminal mass with a transition point.
- An emergency laparotomy with enterotomy successfully removed the mass.
- Psychological evaluation and counseling are essential to prevent recurrence.

Trichobezoars occur most frequently in females under 20 years of age, accounting for nearly 90% of cases, and may present even in very young children^[^2^]^. The stomach is the most common location; however, a trichobezoar may extend into the small bowel (Rapunzel syndrome) or, much more rarely, present as an isolated small intestinal obstruction^[^3^]^. Isolated ileal trichobezoars in children are exceptionally uncommon, with only isolated case reports described in the literature. Trichobezoars are commonly associated with trichotillomania and trichophagia, and around 1% of individuals with trichobezoars require surgical management^[^4^]^. We present a case of a 7-year-old girl with an ileal trichobezoar causing acute intestinal obstruction. This case highlights the diagnostic challenges and the need for a high index of suspicion, even in young children. This report is in accordance with the SCARE Guidelines 2025^[^5^]^.

## Case presentation

A 7-year-old girl presented to the emergency department with a 5-day history of abdominal pain. The pain was acute in onset, generalized, colicky, and non-radiating, with no aggravating or relieving factors. She had multiple episodes of vomiting that were non-projectile, non-bilious, not blood-tinged, and contained food particles. Additionally, she had obstipation for 5 days. There was no history of fever, abdominal trauma, or prior abdominal surgical intervention. On examination, the child was ill-appearing and dehydrated. However, her vitals were stable. The abdomen was distended, with generalized tenderness and guarding, although rebound tenderness was absent. On auscultation, sluggish bowel sounds were heard. Digital rectal examination showed an empty rectum, with no palpable mass and normal anal tone.

Baseline investigations showed a raised total leukocyte count with a neutrophil predominance of 82%; however, the rest of the investigations were within normal limits. The patient was initially resuscitated, and once stabilized, further investigations were pursued. An erect abdominal X-ray revealed a dilated small-bowel loop with multiple air–fluid levels (Fig. 1).

**Figure 1.**
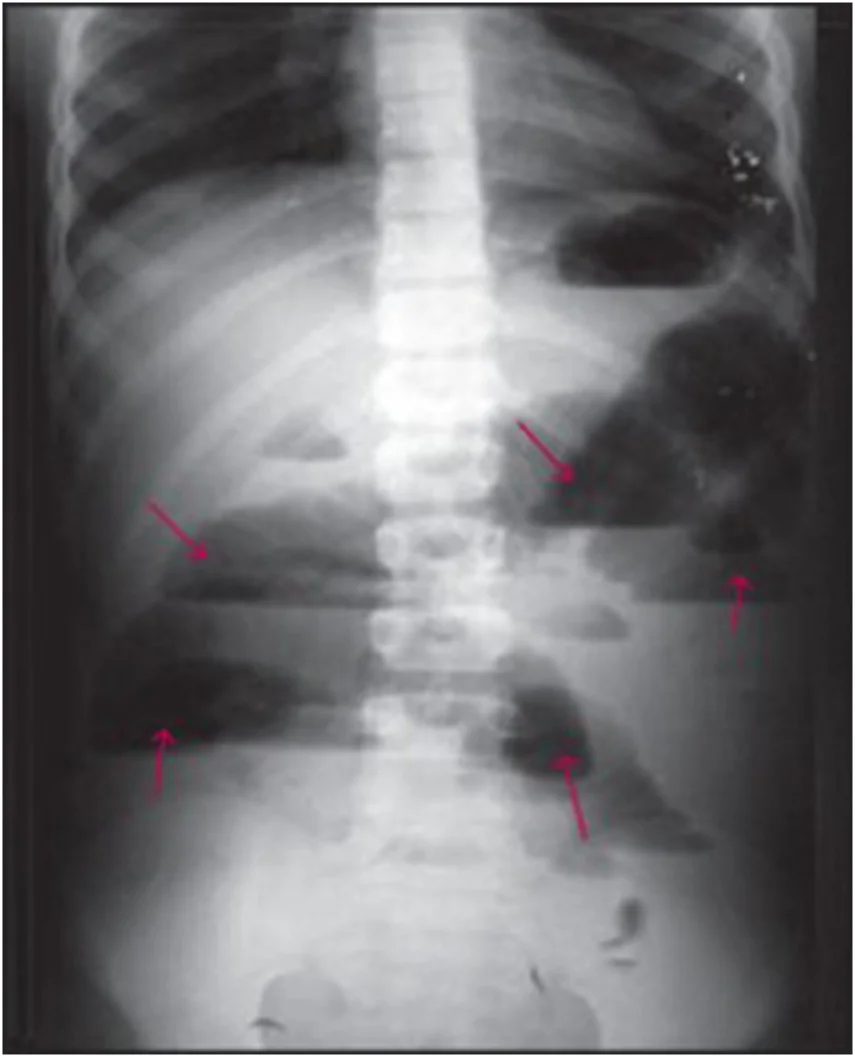
Erect X-ray of the abdomen showing multiple air–fluid levels (arrows indicate multiple air–fluid levels).

In view of acute intestinal obstruction, a CECT of the abdomen was ordered, which showed a large heterogeneous mass of particulate matter representing a trichobezoar, with gastric distension and dilated loops of the small bowel, with an air–fluid level proximal to the particulate debris (Fig. 2A–C).

**Figure 2.**
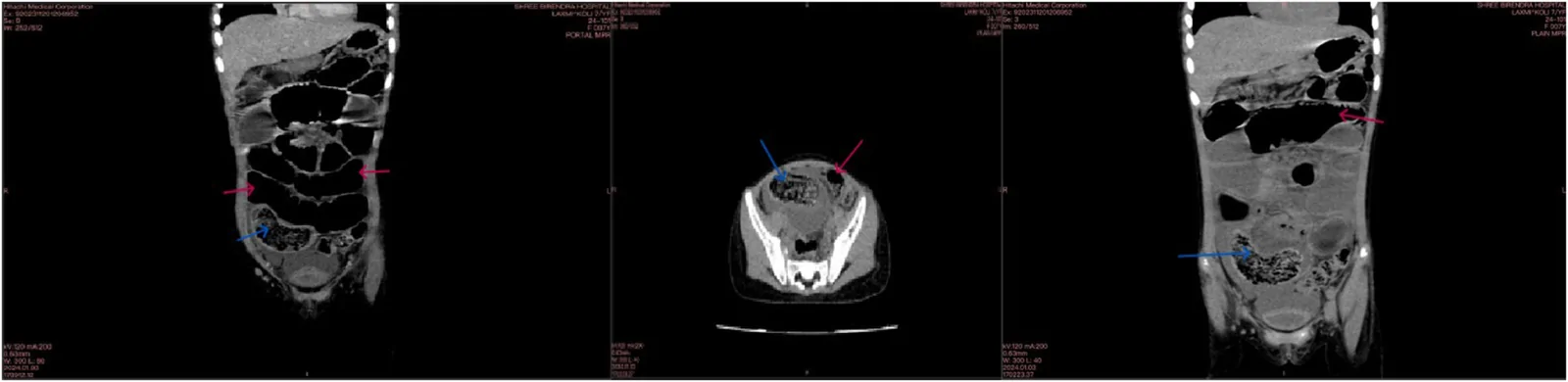
CECT of the abdomen and pelvis showing dilated bowel (red arrow) with particulate debris (blue arrow).

The patient underwent an emergency exploratory laparotomy. Laparotomy was chosen over laparoscopy due to acute obstruction with peritonism, the need for complete bowel palpation to exclude synchronous bezoars, and the safety of open extraction in a young child with abdominal distension. Intraoperative findings revealed dilated proximal small bowel loops and collapsed distal loops. A trichobezoar measuring 9–10 cm in length was removed from the ileum, 20 cm proximal to the ileocecal junction, by enterotomy, and primary closure of the ileum was performed. No additional bezoars were found in the stomach or elsewhere (Fig. 3A–C).

**Figure 3.**
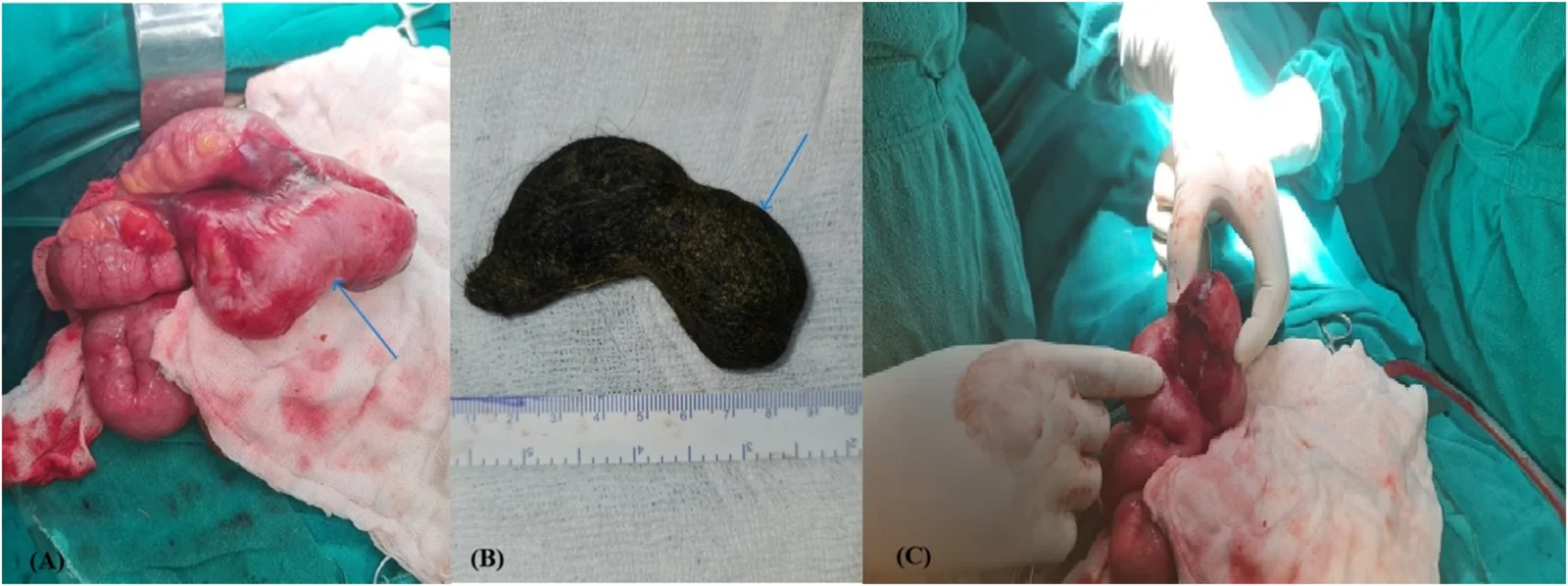
Intraoperative photographs of the procedure. (A) Dilated and congested small-bowel loops with an intraluminal mass causing obstruction (blue arrow). (B) Retrieved trichobezoar specimen (blue arrow). (C) Delivery and extraction of the trichobezoar through an enterotomy in the ileum.

Retrospectively, a history of hair-pulling and ingestion was noted, first observed 2 years earlier. The hairline was found to be receded, with no other areas of alopecia. The child’s postoperative recovery was uneventful. She was discharged on day six, reviewed 2 weeks later, and was asymptomatic with a healed wound. Psychiatric evaluation on postoperative day four confirmed trichotillomania and trichophagia, which were often triggered by parental conflict. Management included cognitive behavioral therapy with habit-reversal training, family therapy, and school-based support. Pharmacotherapy was not required. At the 1-month follow-up, she remained symptom-free with reduced hair-pulling episodes, and at 3 months, there was no clinical evidence of recurrence. Long-term follow-up was planned at 6 and 12 months.

## Clinical discussion

Trichobezoars are rare gastrointestinal foreign bodies composed of ingested hair, most commonly seen in adolescent females with underlying psychiatric or behavioral disorders such as trichotillomania and trichophagia. Approximately one-third of individuals with trichophagia eventually develop trichobezoars^[^6^]^. Risk factors can include depression, anxiety, prior surgeries, and other underlying or associated psychiatric diseases, including post-traumatic stress disorder^[^7^]^.

The smooth surface of hair resists both peristalsis and digestion. As a result, it builds up between the mucosal folds of the stomach. Continuous swallowing of hair over time leads to impaction of hair, along with mucus and food, which results in the formation of a trichobezoar^[^6^]^.

Presentation in younger children, especially those under 10 years of age, is uncommon and often leads to delayed diagnosis due to low clinical suspicion^[^1^]^. The index case highlights the importance of considering bezoars as a differential diagnosis in pediatric patients with prolonged vomiting, abdominal pain, and features of bowel obstruction, particularly when behavioral history may initially be unclear.

Clinical symptoms of trichobezoar-induced obstruction are typically nonspecific, including abdominal pain, distension, nausea, vomiting, early satiety, anorexia, and constipation or obstipation^[^8^]^. Physical examination typically reveals abdominal tenderness or a palpable epigastric mass, though findings may be subtle. Patchy alopecia or evidence of hair-pulling may provide important diagnostic clues; however, such behaviors are frequently unrecognized or denied, as was evident in this case, where the history of trichophagia emerged only retrospectively^[^6^]^.

The complications reported over the years include gastric mucosal erosion, ulceration, and perforation of the stomach or the small intestine, gastric outlet obstruction, intussusception, obstructive jaundice, protein-losing enteropathy, pancreatitis, and death^[^9^]^.

Diagnosis depends on clinical suspicion, supported by imaging. Endoscopy can identify gastric bezoars and allows their removal when they are small and uncomplicated. However, larger bezoars generally require surgical intervention^[^10^]^.

Radiological imaging plays a critical role in diagnosis. Ultrasonography may show dilated bowel loops, but is often limited by gas. Plain abdominal radiographs typically demonstrate small bowel dilatation with multiple air–fluid levels, as seen in this case^[^10^]^. CT scan of the abdomen is the most sensitive modality for detecting bezoar location, size, and associated complications^[^1^]^. In this case, CT findings clearly demonstrated the heterogeneous intraluminal mass and the transition point consistent with a trichobezoar, prompting timely surgical intervention.

Management depends on the size, location, and complications of the bezoar. Endoscopic removal may be attempted for small gastric bezoars; however, success rates remain low for trichobezoars because hair resists chemical digestion and mechanical fragmentation^[^11^]^. In a retrospective review of 108 cases of trichobezoars, only 5% of attempted endoscopic removals were successful, whereas 75% of attempted laparoscopic surgical extractions were successful. However, laparotomy with gastrostomy was 99% successful and thus considered the best management option^[^11^]^. In this patient, emergency exploratory laparotomy with enterotomy allowed successful removal of a 9–10 cm ileal trichobezoar, with excellent postoperative recovery.

Recurrence remains a significant concern, reported in up to 20% of cases^[^6^]^. Preventing recurrence requires addressing the underlying psychiatric or behavioral disorder, as surgical removal alone is insufficient. Comprehensive postoperative care, including psychiatric evaluation, behavioral therapy, counseling, and family education, is essential to reduce relapse risk^[^12^]^. In this case, psychosocial assessment identified a 2-year history of hair-pulling and ingestion, consistent with trichotillomania and trichophagia. Early psychological intervention and continued follow-up were initiated, aligning with recommendations for interdisciplinary long-term management.

## Conclusion

Trichobezoars are an uncommon but important cause of small bowel obstruction in children, particularly when behavioral symptoms are subtle or unrecognized. Timely diagnosis requires high clinical suspicion supported by appropriate imaging. Contrast-enhanced CT is the most definitive modality to identify bezoars and their complications. Surgical removal remains the treatment of choice for distal bezoars causing obstruction. A multidisciplinary approach, including pediatric surgeons, psychologists, and pediatricians, is essential to ensure complete recovery and prevent recurrence.

## Data Availability

Data sharing is not applicable to this article as no new data were created or analyzed in this study.
